# Oxytocin receptors in the Magel2 mouse model of autism: Specific region, age, sex and oxytocin treatment effects

**DOI:** 10.3389/fnins.2023.1026939

**Published:** 2023-03-14

**Authors:** Valentina Gigliucci, Marta Busnelli, Francesca Santini, Camilla Paolini, Alessandra Bertoni, Fabienne Schaller, Françoise Muscatelli, Bice Chini

**Affiliations:** ^1^Institute of Neuroscience, National Research Council, Vedano al Lambro, Italy; ^2^NeuroMI Milan Center for Neuroscience, University of Milano-Bicocca, Milan, Italy; ^3^Department of Medical Biotechnology and Translational Medicine, Università degli Studi di Milano, Milan, Italy; ^4^Aix Marseille University, INSERM, INMED, Marseille, France

**Keywords:** neurodevelopmental disorders (NDD), Schaaf-Yang Syndrome, Prader-Willi Syndrome (PWS), postnatal oxytocin treatment, oxytocin receptor expression

## Abstract

The neurohormone oxytocin (OXT) has been implicated in the regulation of social behavior and is intensively investigated as a potential therapeutic treatment in neurodevelopmental disorders characterized by social deficits. In the *Magel2*-knockout (KO) mouse, a model of Schaaf-Yang Syndrome, an early postnatal administration of OXT rescued autistic-like behavior and cognition at adulthood, making this model relevant for understanding the actions of OXT in (re)programming postnatal brain development. The oxytocin receptor (OXTR), the main brain target of OXT, was dysregulated in the hippocampus of *Magel2*-KO adult males, and normalized upon OXT treatment at birth. Here we have analyzed male and female *Magel2*-KO brains at postnatal day 8 (P8) and at postnatal day 90 (P90), investigating age, genotype and OXT treatment effects on OXTR levels in several regions of the brain. We found that, at P8, male and female *Magel2*-KOs displayed a widespread, substantial, down-regulation of OXTR levels compared to wild type (WT) animals. Most intriguingly, the postnatal OXT treatment did not affect *Magel2*-KO OXTR levels at P8 and, consistently, did not rescue the ultrasonic vocalization deficits observed at this age. On the contrary, the postnatal OXT treatment reduced OXTR levels at P90 in male *Magel2*-KO in a region-specific way, restoring normal OXTR levels in regions where the *Magel2*-KO OXTR was upregulated (central amygdala, hippocampus and piriform cortex). Interestingly, *Magel2*-KO females, previously shown to lack the social deficits observed in *Magel2*-KO males, were characterized by a different trend in receptor expression compared to males; as a result, the dimorphic expression of OXTR observed in WT animals, with higher OXTR expression observed in females, was abolished in *Magel2*-KO mice. In conclusion, our data indicate that in *Magel2*-KO mice, OXTRs undergo region-specific modifications related to age, sex and postnatal OXT treatment. These results are instrumental to design precisely-timed OXT-based therapeutic strategies that, by acting at specific brain regions, could modify the outcome of social deficits in Schaaf-Yang Syndrome patients.

## Introduction

Oxytocin (OXT) is a small neuropeptide released by the hypothalamus into the bloodstream to control lactation and parturition and in the brain to control several aspects of behavior, such as emotional and social processing (Jurek and Neumann, 2018).

The action of OXT within the brain is mediated by OXT binding to a specific receptor, the oxytocin receptor (OXTR) (Busnelli and Chini, 2018). In some conditions, for example in the presence of supraphysiological OXT concentrations, OXT can also activate the highly related vasopressin 1a and 1b receptors (V1aR and V1bR) (Chini et al., 2017). OXTR is a G-protein coupled receptor expressed in several areas of the brain, and one of its most compelling features is the extreme variability in its regional distribution within the brain, which has been shown to be linked to a number of factors, including the species, sex and developmental age, as well as several environmental influences.

A highly variable regional distribution of OXTR is observed in mammals, even between closely-related species (Walum and Young, 2018), originally described in the prairie and montane voles, where different OXTR distributions relate to striking differences in social behavior (Insel and Shapiro, 1992; Young and Wang, 2004). Region-specific sex differences have also been reported in different species including mice (Hammock and Levitt, 2013; Sharma et al., 2019; Newmaster et al., 2020). Age is another well-established factor influencing OXTR levels, and the OXTR distribution in the brain undergoes dynamic changes in expression through the postnatal development, with peak expression at early infancy in rodents and humans (Tribollet et al., 1989; Hammock and Levitt, 2013; Vaidyanathan and Hammock, 2017; Prounis et al., 2018; Newmaster et al., 2020).

One relevant issue in the field is how OXT and OXTR levels in the early postnatal life influence the development of social abilities during infancy, adolescence and adult life and, most importantly, how these levels are modulated. A large body of literature, pioneered by Karen Bales and co-authors, has clearly shown that early-life experience has long-term effects on the OXT system, including the expression of OXTR (Bales and Perkeybile, 2012; Veenema, 2012; Perkeybile et al., 2019; Lapp et al., 2020). Mechanistically, it has been shown that environmental factors, particularly during early infancy, can epigenetically modify the OXTR gene and influence its expression levels at adulthood (Carter et al., 2020; Onaka and Takayanagi, 2021).

Early modulation of OXTR levels is of particular importance when considering neurodevelopmental disorders, many of which are characterized by deficits in social abilities and social cognition e.g. autism spectrum disorders (ASD) and schizophrenia. Several mouse models of neurodevelopmental disorders present abnormalities in OXT release and/or OXTR distribution (Meziane et al., 2015; Wagner and Harony-Nicolas, 2018; Borie et al., 2021), providing a strong rationale for the use of OXT as a possible therapeutic agent.

The *Magel2*-knockout (*Magel2*-KO) mouse has proven to be extremely useful to study the role of OXT and OXTR in a mouse model presenting neurodevelopmental impairments (Fountain and Schaaf, 2015; Meziane et al., 2015; Muscatelli et al., 2018). These mice lack *Magel2*, a gene contained in the Prader-Willi Syndrome (PWS) locus, an imprinted chromosomal region also known as “PWS paternal-only expressed region” (Butler et al., 2019). In humans, the specific lack of expression of the *MAGEL2* gene, causes a Prader Willi-like disease identified as Schaaf-Yang Syndrome (SYS; OMIM 615547) (Schaaf et al., 2013). In addition to many pathological PWS phenotypic traits, such as neonatal hypotonia, hypogonadism and feeding problems, this syndrome also presents a higher prevalence of autism spectrum disorders (up to 75% of affected individuals) (Schaaf and Marbach, 1993, updated in 2021).

*Magel2*-deficient mice recapitulate autistic-like symptoms and other defects observed in SYS patients. In particular, neonate *Magel2*-KO mice show feeding defects due to an altered onset of suckling activity, leading to neonatal growth retardation and a high mortality rate (approx. 50%) (Schaller et al., 2010); feeding defects are accompanied by low rates of separation-induced vocalizations and altered spectral features (Bosque Ortiz et al., 2022). The surviving adult *Magel2*-KO mice have been extensively investigated for alterations in sensory-motor, cognitive and social abilities. No differences were found between WT and *Magel2*-KO female mice, while altered spatial learning and social recognition memory were found in male *Magel2*-KO mice (Meziane et al., 2015; Bertoni et al., 2021). A reduction in mature OXT, with the accumulation of intermediate forms of the peptide, was reported in the neonate *Magel2*-KO hypothalamus, suggesting an impaired processing of the prohormone (Meziane et al., 2015); in contrast, a significant increase (2-fold) of mature OXT was found in the hypothalamic-hypophyseal system of adult *Magel2*-KO mice, accompanied by an increased number of OXT-expressing neurons (Meziane et al., 2015), representing a possible compensatory mechanism to overcome the strong suppression of the electrophysiological activity observed in OXT-expressing neurons (Ates et al., 2019). Most importantly, an early postnatal OXT treatment was demonstrated to rescue neonatal lethality and to prevent the appearance of social and learning deficits in adult *Magel2-*KO mice (Meziane et al., 2015; Bertoni et al., 2021), providing strong preclinical evidence for pilot studies of OXT treatment in PWS and SYS infants, such as that conducted in PWS babies showing encouraging positive results (Tauber et al., 2017).

More recently, a detailed investigation of the molecular bases of social memory impairments in *Magel2-*KO males revealed specific alterations in hippocampal circuitry and functions (Bertoni et al., 2021). In particular, *Magel2-*KO adult mice display an increased OXTR expression in the dorsal CA2/CA3 (dCA2/CA3) and in the Dentate gyrus (DG), but not in the ventral vCA2/CA3 region (vCA2/CA3) of the hippocampus. Moreover, postnatal OXT normalized OXTR in DG, but not in the dCA2/CA3 region (Bertoni et al., 2021). These findings strongly suggest that region specific alterations in receptor expression are present in *Magel2*-KO mice, and that postnatal OXT treatment could modulate OXTR in specific brain regions.

In the present work we extended the regional mapping of OXTR in male and female *Magel2*-KO brains, with or without treatment of OXT during the first week of postnatal life. As OXTRs are strongly regulated in mice in the first three weeks after birth (Hammock and Levitt, 2013; Newmaster et al., 2020), we investigated if the OXT treatment received in the first week of life had short and/or long term impact on regional OXTR levels. Understanding the specific sites within the brain where OXT exerts its rescue action is a fundamental step to strengthening the rationale for its use in PWS/SYS and further neurodevelopmental disorders.

We also evaluated the sexual dimorphism of OXTR distribution in *Magel2*-KO mice and looked for sex-specific modulation of OXTR by postnatal OXT treatment. Autism-related disorders are characterized by a strong sex bias, with a male to female ratio among affected individual close to 4:1 (Ferri et al., 2018). Understanding the molecular basis of sex differences could contribute to understanding the fundamental mechanisms of the biology of autism itself.

## Materials and methods

### Animals

*Magel2* ^tm1.1Mus +/+^ (referred to as WT) and *Magel2* ^tm1.1Mus –/–^ (referred to as *Magel2*-KO) mice (Bertoni et al., 2021) were maintained on a C57BL/6J genetic background and housed in standard conditions, with *ad libitum* access to food and water. Mice were handled and cared for in accordance with the Guide for the Care and Use of Laboratory Animals (N.R.C., 2011) and the European Communities Council Directive of September 22 2010 (2010/63/EU, 74). All the experimental procedures were approved by the French Ministry of Agriculture with the accreditation no. B13-055-19. The protocol included 6 experimental groups (Group 1-6) for brain autoradiography, each composed of 3 males and 3 females, for a total of 36 animals and 3 experimental groups (Group 7-9) for ultrasonic vocalization (USVs) analysis, each composed of males and females, for a total of 100 animals, 53 males and 47 females.

Group 1: WT mice treated with saline and sacrificed at P8; Group 2: WT mice treated with saline and sacrificed at P90; Group 3: *Magel2-*KO mice treated with saline and sacrificed at P8; Group 4: *Magel2*-KO mice treated with saline and sacrificed at P90; Group 5: *Magel2-*KO mice treated with OXT and sacrificed at P8; Group 6: *Magel2-*KO *mice* treated with OXT and sacrificed at P90; Group 7: WT mice treated with saline and tested for USVs at P8; Group 8: *Magel2*-KO mice treated with saline and tested for USVs at P8; Group 9: *Magel2*-KO mice treated with OXT and tested for USVs at P8. The day of birth was considered postnatal day 0 (P0).

A *post-hoc* power calculations was performed on the autoradiography datasets recently published (Bertoni et al., 2021). Such analyses was run with the G*Power 3.1 software (RRID:SCR_013726, University of Düsseldorf).^1^ The effect size f was derived from the available datasets (1.83 for DG and 1.66 for dCA2/CA3), and transferred into a *post-hoc* ANOVA (Fixed effects, omnibus, one-way) test with set number of groups = 3, total sample size = 9 and alpha error = 0.05. We then calculated the effective power achieved in Bertoni et al. (2021) for the DG and the dCA2/CA3, which were 0.97 and 0.93, respectively, thus indicating that 3 mice/group are sufficient for this type of analysis.

### Treatments

**FIGURE 1:**
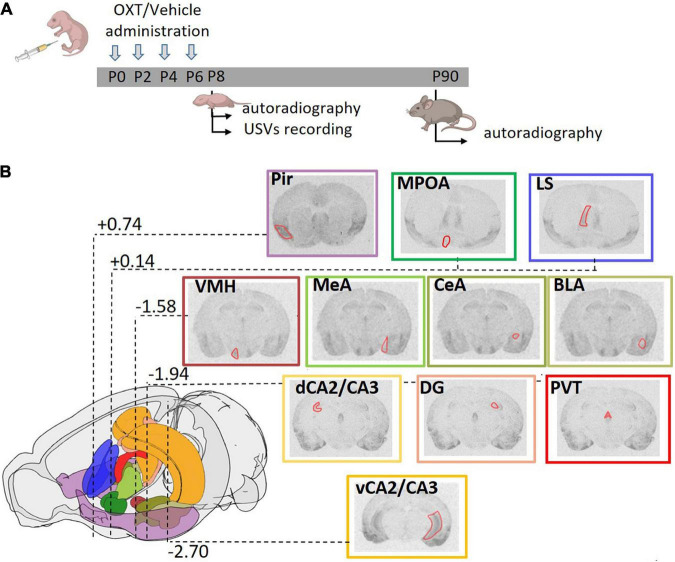
Experimental strategy. **(A)** Schematic diagram representing the treatment regime administered to the mice and the paradigm of the analysis performed. Mice were subcutaneously injected with OXT or vehicle (single injection/day) in the first week of life at P0, P2, P4, and P6. Brain autoradiography was performed at P8 or P90. A separate group of animals at P8 was tested for ultrasonic vocalizations (USVs). **(B)** Schematic representation of the mouse brain showing the stereotactic coordinates of the coronal planes in which OXTR were analyzed. Distance from bregma, reported in millimeters according to Franklin and Paxinos mouse brain atlas (Franklin and Paxinos, 2007), are highlighted for the most representative section. A color code was used to identify the different areas analyzed: violet: Pir, piriform cortex; emerald green: MPOA, medial preoptic area; blue: LS, lateral septum; brown: VMH, ventral medial nucleus of the hypothalamus; three values of olive green for MeA, medial amygdala; CeA, central amygdala; BLA, basolateral amygdala; orange: dCA2/CA3, dorsal and vCA2/CA3, ventral field CA2 and CA3 of the hippocampus; pink: DG, dentate gyrus; red: PVT, paraventricular thalamic nucleus. Within each representative autoradiographic section, whose border color follows the color code reported above, the ROI used for acquisition of the data is depicted in red.

Three to five hours after delivery, pups were subcutaneously injected with saline or OXT (Phoenix Pharmaceuticals. Inc., Strasbourg, France; Catalog No.051-01) dissolved in isotonic saline at a final concentration of 0.2 μg/μl (20 μl/injection). Four administrations of OXT or saline (indicated as Vehicle and abbreviated to Veh) were given subcutaneously every 2 days (at P0, P2, P4, and P6) to male and female *Magel2-*KO mice immediately after birth (Figure 1A). The dose and administration timings were selected on the basis of previous work showing that this administration protocol prevented social and learning deficits in adult *Magel2*-KO mice (Bertoni et al., 2021).

### Brain collection and slice preparation

At P8 or P90, mice were sacrificed, the brains quickly extracted, flash-frozen by dipping in cold (-25°C) isopentane (Sigma Aldrich) and stored at −80°C until processing. 14 um coronal sections were collected using a Frigocut-2700 (Reichert-Jung) cryostat and collected on gelatin/chromium potassium sulfate-coated Superfrost slides.

### Receptor autoradiography and ROI analysis

Quantification of OXTR was performed by receptor autoradiography according to the protocol originally developed by E. Tribollet (Tribollet et al., 1989). Brain sections were lightly fixed by dipping the slides for 5 min in a solution of 0.2% paraformaldehyde in 0.1 M phosphate-buffered saline (pH 7.4), then rinsed twice in 50 mM Tris–HCl buffer (pH 7.4) supplemented with 0.1% bovine serum albumin. Incubation was carried out for 2 hr under gentle agitation at room temperature in a humid chamber (kindly donated by E. Tribollet) by covering each slide with 400 μl incubation medium (50 mM Tris–HCl, 0.1 mM bacitracin, 5 mM MgCl_2_, 0.1% bovine serum albumin) containing 0.02 nM final concentration of radioiodinated OXTR antagonist [^125^I]d(CH_2_)_5_[Tyr(Me)^2^,Thr^4^,Tyr^9^-NH_2_]OVT (^125^I-OVTA), Perkin Elmer, MA, USA (Elands et al., 1988a,b). The specific activity of radioligand was 2200 Ci/mmol; 1Ci = 37GBq. Non-specific binding was evaluated by incubating adjacent sections with incubation medium containing 0.02 nM ^125^I-OVTA and 2 μM OXT. Incubation was followed by two 5 min washes in ice-cold incubation medium and a quick rinse in distilled water. The slides were rapidly dried under a stream of cool air. Once dry, the slides were placed in an X-ray cassette in contact with Biomax MR Films (Carestream, USA, #891–2560). After 5 days of exposure each film was developed and scanned for image analysis.

Regions of Interest (ROIs) were designed using the Franklin and Paxinos’ Mouse Brain Atlas (Franklin and Paxinos, 2007) as reference. Regions analyzed include: the piriform cortex (Pir), the medial preoptic area (MPOA), the lateral septum (LS), the ventromedial nucleus of the hypothalamus (VMH), the amygdala in its basolateral (BLA), medial (MeA), and central (CeA) subdivisions, the hippocampus in its dorsal (dCA2/CA3), ventral (vCA2/CA3) and Dentate Gyrus (DG) regions and the paraventricular nucleus of the thalamus (PVT) (Figure 1B). Densitometric gray level were measured using NIH ImageJ software. To obtain single ROI values, the gray level of the film (corresponding to background) was subtracted to the gray level of a ROI on a slice incubated with ^125^I-OVTA (corresponding to total binding) and to the gray level of a ROI on an adjacent slice incubated with ^125^I-OVTA + OXT (corresponding to non-specific binding); the obtained densitometric gray level values of total and non-specific binding were then converted to nCi/mg tissue equivalent using an autoradiographic iodinated ^125^I-microscales (Amersham) on films exposed for 5 days (kindly provided by E. Tribollet). The specific binding value of each ROI was finally obtained by substracting non-specific binding to total binding. For each animal, at least 3 ROIs for each region were acquired (adjacent slices and/or left and right hemisphere areas on the same slice for bilateral regions).

### Ultrasonic vocalization (USVs)

Briefly, the mother and litter were left to habituate to the testing room for 30 min, then P8 pups were separated from the mother and gently transferred to a new cage on a heating pad (37°C). After 5 min, each pup was transferred in an anechoic box (54 × 57 × 41 cm; Coulbourn instruments, PA, USA) and USVs were immediately recorded for 300 s by an ultrasonic microphone (Avisoft UltraSoundGate condenser microphone capsule CM16/CMPA, Avisoft bioacustic, Germany) sensitive to frequencies of 10-250 kHz. Recordings were done using Avisoft recorder software (version 4.2) with a sampling rate of 250 kHz in 16-bit format. Data were analyzed for the total number of calls using Avisoft SASLab software.

### Statistical analysis

All graphs have been created and datasets analyzed with GraphPad Prism ver. 8.0.2 (GraphPad Software, Inc.). Molecular data were analyzed by analysis of variance (ANOVA). In particular, for the combined analysis of age, sex and genotype, for each brain area a three-way ANOVA was applied, followed by Tukey’s multiple comparisons *post-hoc* test. Similarly, for the analysis by region of the combination of age, sex and OXT treatment, three-way ANOVA was applied, followed by Tukey’s multiple comparisons *post-hoc* test. For treatment efficacy, two-way ANOVA followed by Tukey’s multiple comparisons *post-hoc* test was applied, to account and correct for multiple testing. Adjusted *p*-values of *p* < 0.05 were deemed significant. USVs data were analyzed by One-way ANOVA followed by Tukey’s multiple comparisons *post-hoc* test. Significance was set at *p*-values of *p* < 0.05. Bars in the graphs display values as mean ± SEM.

**FIGURE 2:**
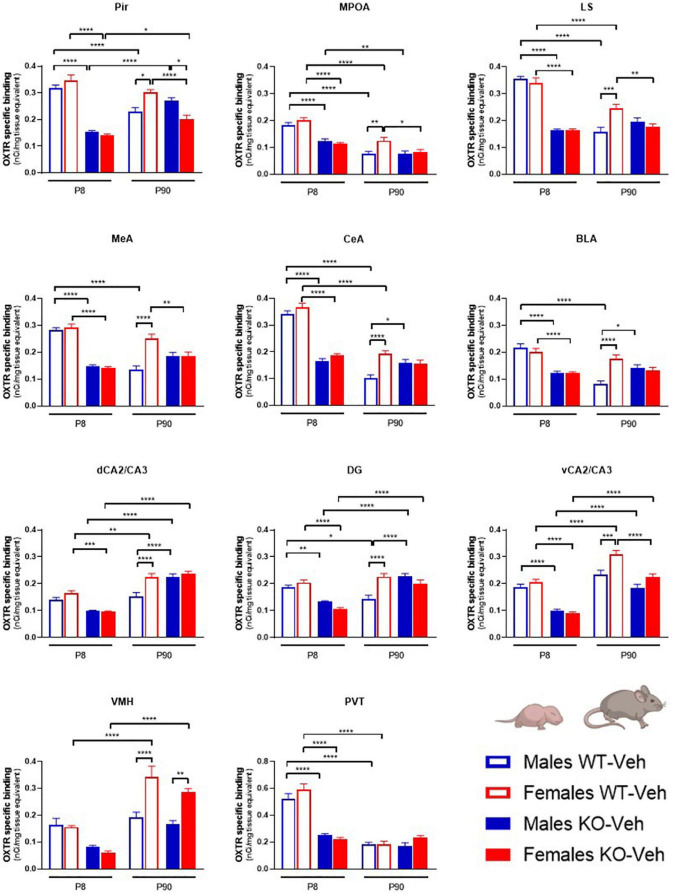
Contributions of age, sex and Magel2-KO genotype on physiological brain regional OXTR expression levels. Bar graphs of OXTR levels quantified by [^125^I]-OVTA binding in P8 and P90, male and female, WT and *Magel2-KO* mice. Each histogram represents data expressed as mean + SEM of multiple datapoints collected from three animals. Unfilled bars are used for vehicle (Veh) treated WT, filled bars are used for vehicle (Veh) treated *Magel2-KO*; blue is used for males of both genotypes and ages, red for females of both genotypes and ages. Data were analyzed by three-way ANOVA, followed by a Tukey’s multiple comparisons *post-hoc* test. *p < 0.05, **p < 0.01, ***p < 0.001, ****p < 0.0001. When a comparison was approaching statistical significance, the corresponding *p*-value was reported on the appropriate graph. Datasets and detailed statistical analyses are reported in the Supplementary Table 1.

**FIGURE 3:**
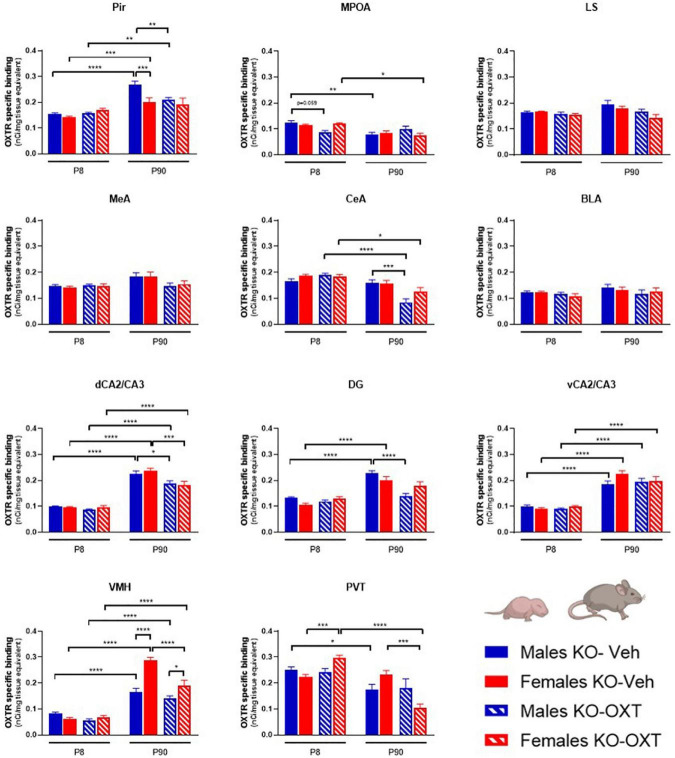
Long-lasting effects of a postnatal OXT on brain regional OXTR expression levels in Magel2-KO mice. Bar graphs of OXTR levels quantified by [^125^I]-OVTA binding in P8 and P90, male and female, Vehicle or OXT-treated *Magel2-KO* mice. Each histogram represents data expressed as mean + SEM of multiple datapoints collected from three animals. Filled bars are used for vehicle (Veh) treated *Magel2-KO*, striped bars are used for oxytocin (OXT) treated *Magel2-KO*; blue is used for males of both treatment groups and ages, red for females of both treatment groups and ages. Data were analyzed by Three-way ANOVA, followed by a Tukey’s multiple comparisons *post-hoc* test. *p < 0.05, **p < 0.01, ***p < 0.001, ****p < 0.0001. When a comparison was approaching statistical significance, the corresponding *p*-value was reported on the appropriate graph. Datasets and detailed statistical analyses are reported in the Supplementary Table 2.

**FIGURE 4:**
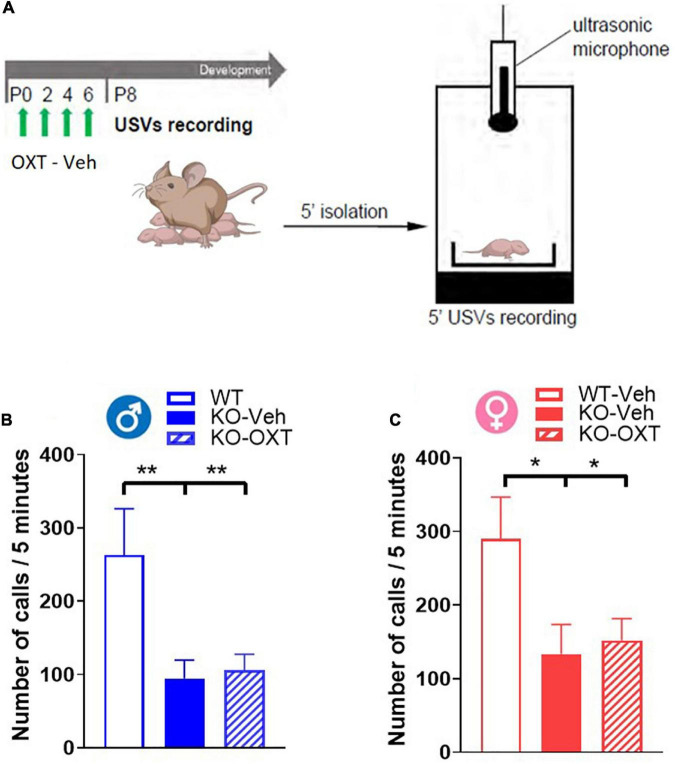
Ultrasonic vocalization calls (USVs) in P8 male and female WT and Magel2-KO pups treated with vehicle or OXT during the first week of life. **(A)** Schematic drawing of the protocol used to record separation-induced USVs in P8 mice. Pups were treated with vehicle (Veh) or oxytocin (OXT) from P0 to P6. Number of total calls, measured during 5 min isolation after pup separation in males **(B)** and females **(C)**. Each histogram represents data expressed as mean + SEM of 11-24 mice. Unfilled bars are used for vehicle (Veh) treated WT; filled bars are used for vehicle (Veh) treated *Magel2-*KO; striped bars correspond to oxytocin (OXT) treated Magel2-KO; blue bar is used for males of both genotypes, red bar for females of both genotypes. Histograms indicate the mean + SEM of the different groups analyzed by one-way ANOVA followed by a Tukey’s multiple comparisons *post-hoc* test. *p < 0.05, **p < 0.01. Values and statistics are reported in Supplementary Table 3.

**FIGURE 5:**
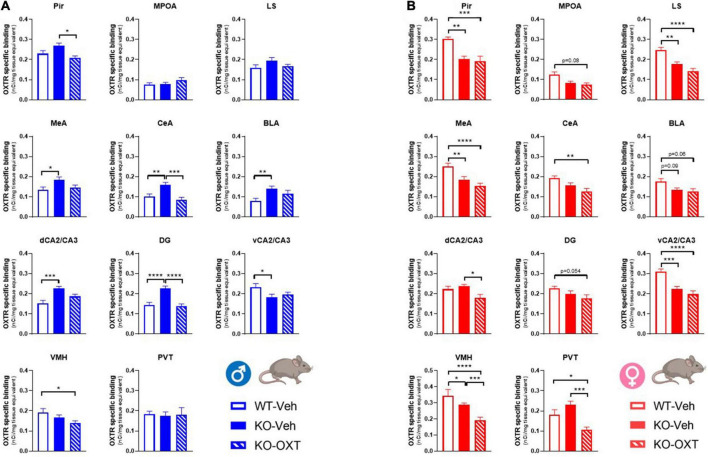
Long term effects of the postnatal OXT treatment on brain OXTR expression levels in P90 male and female WT and Magel2-KO mice. Bar graphs of OXTR levels quantified by [^125^I]-OVTA binding in adult male **(A)** and female **(B)** mice. Each histogram represents data expressed as mean + SEM of multiple datapoints collected from 3 animals. Unfilled bars are used for vehicle (Veh) treated WT, filled bars are used for vehicle (Veh) treated *Magel2*-KO, striped bars are used for oxytocin (OXT) treated *Magel2*-KO. Data were analyzed by two-way ANOVA, followed by a Tukey’s multiple comparisons *post-hoc* test. *p < 0.05, **p < 0.01, ***p < 0.001, ****p < 0.0001. When a comparison was approaching statistical significance, the corresponding *p*-value was reported on the appropriate graph. Datasets and detailed statistical analyses are reported in the Supplementary Tables 4A, B.

The complete analysis of the all statistical data included in Figures 2-5 is reported in the Supplementary Tables 1-4.

## Results

We have previously found a dysregulation of OXTRs in the hippocampus of male *Magel2*-KO mice that was normalized at adulthood by postnatal OXT treatment (Bertoni et al., 2021). Here, as outlined in Figure 1A, we designed a new study which also included female mice and the analysis of other brain regions relevant for OXT actions (reported in Figure 1B). *Magel2-*KO mice were treated during the 1*^st^* postnatal week and OXTR brain expression was analyzed at an early postnatal developmental stage (P8) and in adults (P90) (Figures 1A, B).

The possible interaction between sex, genotype and age in determining OXTR brain expression variations was analyzed in male and female WT and *Magel2-*KO mice at P8 and P90. A three-way ANOVA analysis was performed on OXTR levels measured in WT and *Magel2*-KO brains postnatally treated with saline. All statistical data of time x sex x genotype analysis are reported in the Supplementary Table 1.

As shown in Figure 2, we found that, at P8, OXTR expression in *Magel2*-KO brains was significantly reduced in all regions analyzed as compared to control WT animals, in both males and females; in only two regions, the trend toward a reduction did not reach a statistically significant value (male dCA2/CA3 hippocampus *p* = 0.052; males VMH *p* = 0.26 and females VMH *p* = 0.079). At this developmental stage, male and female WT animals expressed similar levels of OXTR in all areas, and, similarly, no sexually dimorphic areas for OXTR expression were found at P8 in *Magel2*-KO animals.

At P90, as compared to WT, adult *Magel2-*KO males displayed up-regulated OXTR levels in the amygdala (CeA and BLA) and in the hippocampus (dCA2/CA3 and DG). In contrast, a down-regulation of OXTR levels was observed in *Magel2*-KO females in the Pir, MPOA, LS, MeA and vCA2/CA3. At this age, in WT animals, a significant sexual dimorphic OXTR expression, with higher levels in females, was observed in all regions investigated with the only exception of the PVT. In *Magel2*-KO mice, male/female differences were only present in the Pir (where higher OXTR levels were measured in males) and in the VMH (where higher OXTR levels were observed in females); in all the other regions investigated *Magel2*-KO mice lost the sexual dimorphism in OXTR levels.

Finally, the analysis of the OXTR expression by genotype at the two different ages (P8 and P90) indicated a widespread age-dependent reduction of OXTR levels in WT animals, particularly in males. The only regions in which OXTR remained elevated in adult males were the hippocampus (dCA2/CA3 and vCA2/CA3) and the VMH. Notably, these are the same regions in which a substantial up-regulation was observed at P90 in WT female brains. In contrast, in *Magel2*-KO male mice, OXTR levels at P90 did not decrease as compared to the levels observed at P8, but remained stable (LS, MeA, CeA, BLA, VMH, and PVT) or, in some cases, increased (Pir, vCA2/CA3, DG and dCA2/CA3); the only region in which a reduction was observed was the MPOA; a similar trend was also observed in the *Magel2*-KO female mice. These results indicate a substantial alteration in the postnatal developmental patterns of OXTR expression in the *Magel2*-KO mice that presented low levels of OXTR at P8 and failed to reach physiological OXTR levels at P90.

We then investigated if postnatal OXT treatment could normalize the altered OXTR levels observed in *Magel2*-KO mice. Figure 3 reports the results of a three-way ANOVA analysis of OXTR binding levels in male and female *Magel2*-KO brains at P8 and P90 treated with OXT or Veh. All statistical data of time x treatment x sex analysis are reported in Supplementary Table 2.

With respect to OXT treatment, no differences were observed at P8 in any of the investigated areas in *Magel2*-KO males and females, with the only exception of a down-regulation of OXTR in male MPOA and an up-regulation of OXTR in female PVT. These results indicate that the postnatal administration of OXT (with the administration schedule used here) had no widespread short-term effects on OXTR levels in the *Magel2*-KO brain at this particular developmental stage.

In adult *Magel2*-KO mice, OXT treatment down-regulated OXTR levels in selected regions in males (Pir, CeA, dCA2/CA3, DG) and females (dCA2/CA3, VMH, and PVT), with no significant effects in the other areas.

Not finding any acute effect of OXT on OXTR levels in *Magel2*-KO pups at P8, we decided to assess if this lack of effect was also observed on a behavioral test that can be performed at this early postnatal developmental stage. Mother separation-induced USVs in pups are used as measure of early communication behavior in rodents and *Magel2-*KO pups have been previously reported to exhibit low rates of separation-induced vocalization with altered spectral features (Bosque Ortiz et al., 2022). We thus assessed the effect of an early OXT treatment on USVs in vehicle-treated *Magel2*-KO and vehicle-treated WT mice at P8 (Figure 4A) and observed lower rates of separation-induced USVs in both male and female *Magel2*-KO (KO-Veh) pups than in WT mice (Figures 4B, C), confirming the results previously obtained (Bosque Ortiz et al., 2022). Our data showed no effect of the perinatal OXT treatment on USV calls in either male or female *Magel2*-KO pups (Figures 4B, C), indicating that this impaired behavior cannot be acutely rescued by postnatal OXT treatment (Supplementary Table 3).

Finally, it was important to assess if the postnatal treatment in *Magel2*-KO had successfully restored OXTR levels similar to those observed in WT mice. In Figure 5, we report a multiple comparison analysis between WT mice treated with vehicle, *Magel2*-KO animals treated with vehicle and *Magel2*-KO treated with OXT. All statistical data analyses are reported in Supplementary Tables 4A, B. Representative film images of the various brain areas displaying main effects of treatment, genotype and sex are reported in Supplementary Figure 1.

Our data indicate that in *Magel2*-KO males, the postnatal OXT treatment reduced OXTR levels in all areas in which the receptor was abnormally upregulated (CeA, MeA, BLA, dCA2/CA3, DG) as well as in the Pir and VMH (Figure 5A and Supplementary Figure 1).

In *Magel2*-KO females, which display similar (MPOA, CeA, BLA, dCA2/CA3, DG, and PVT) or reduced (Pir, LS, MeA, vCA2/CA3, and VMH) OXTR levels compared to WT, the OXT treatment either had no effect (Pir, MPOA, LS, MeA, CeA, BLA, dCA2/CA3, and DG) or induced a further down-regulation of OXTR levels (VMH and PVT) below those observed in WT animals.

In CeA, dCA2/CA3, PVT as compared to those observed in WT females, the final effect of the OXT was a down-regulation of OXTR levels below those observed in WT animals (Figure 5B).

## Discussion

Our data indicate that highly variable region-specific patterns of OXTR expression related to age, sex and postnatal OXT treatment characterize male and female *Magel2*-KO mice.

### OXTR levels at P8 in WT and *Magel2*-KO mice

The most striking result at P8 in *Magel2*-KO mice is the significant reduction in OXTR levels in all the regions analyzed when compared to WT animals, indicating a major defect in OXTR expression trajectories at this very early postnatal developmental age. Equally relevant are the findings that this reduction is observed in male and female animals and that no short-term effect of OXT treatment is evident, again, in either males or females.

A transient, developmental remodeling of OXTR expression has been previously reported in the male mouse brain (Hammock and Levitt, 2013), revealing a peak of OXTR expression in the 2^nd^ postnatal week, which parallels maximal synaptogenesis and experience-dependent plasticity. A more recent study mapped the pattern of postnatal OXTR expression through the brain and confirmed a progressive increase in OXTR expression, which reached a peak in the 3^rd^ postnatal week of life and decreased toward young adulthood (Newmaster et al., 2020). This general developmental profile is observed in a number of rodent species, despite regional differences that possibly subtend species-specific behavioral and/or developmental features: in rats, OXTRs were higher in juveniles than in adults in regions associated with reward and socio-spatial memory, while the opposite was found in regions of the social decision-making network (Smith et al., 2017). Our data confirm a generalized decreased OXTR binding in adult WT animals; in contrast, in the majority of the areas analyzed, P8 *Magel2*-KO mice display receptor levels similar to the low levels observed in the WT adults.

A key question concerns the mechanism(s) responsible for these low OXTR levels in *Magel2*-KO animals at this early developmental age. Our current data indicate that the reduction of OXTR levels affects several regions of the brain, at least all the ones analyzed for this work, therefore we favor the hypothesis of a mechanism based on a global impairment of OXTR expression at the protein and/or mRNA levels. As reduced OXT release throughout the brain could result in a general reduction in OXTR levels (Kenkel et al., 2019), one possibility is that a reduced neonatal OXT release could be responsible for a generalized down-regulation of OXTR. Indeed, reduced OXT production in the *Magel2-*KO neonate brain has been previously reported (Meziane et al., 2015). *Magel2* belongs to the *MAGE* family of ubiquitin ligase regulators, and has been shown to be involved in ubiquitination, actin regulation and endosomal sorting processes; in particular, it might regulate the quantity of secretory granules and bioactive neuropeptide production (Chen et al., 2020). *Magel2* is highly expressed in the hypothalamus during development, and, in the adult brain, high levels of *Magel2* mRNA are found in the neurons of suprachiasmatic (SCN), paraventricular (PVN), and supraoptic (SON) nuclei of the hypothalamus, strongly supporting an implication in OXT storage and release. Other mechanisms could contribute to variations in OXTR levels independently from OXT release: for instance, in the hippocampus, *Magel2* and OXTR mRNAs are co-expressed, as shown by RNAScope experiments (Bertoni et al., 2021), and a *Magel2* deficiency could directly down-regulate the quantity of OXTRs at the cell membrane by affecting the intracellular recycling of membrane proteins.

Quite surprisingly, our data show that postnatal administration of OXT has no short-term effects on OXTR levels in the *Magel2*-KO neonate brain and, in parallel, on the low rate of USV calls that characterize *Magel2*-KO pups. These findings suggest that the effects of OXT on the adult brain depend on long lasting regulation of critical neuronal functions, in agreement with the proposal that life-long OXTR expression is, to some extent, determined by early life OXT exposure, a hypothesis known as “hormonal imprinting” effect (Carter, 2003; Kenkel et al., 2019). Neuronal and astrocytic differentiation/maturation, as well as microglia neurodevelopmental effects, continue in mice until the end of the first month of life, therefore trajectories of neuronal network plasticity and connectivity can be modulated by postnatal OXT with effects extending into adulthood, as elegantly demonstrated in the developing sensory cortices subjected to sensory deprivation (Zheng et al., 2014). In the early postnatal period OXT has been shown to play a role in the establishment of a correct excitation/inhibition (E/I) balance via modulation of the timing of the GABA switch (Leonzino et al., 2016; Ben-Ari, 2018), a decisive time point in neurodevelopmental trajectories that regulates proliferation, migration, differentiation and plasticity of developing neurons and whose perturbation is linked to a number of disorders (Virtanen et al., 2021). Consistently, in *Magel2*-KO hippocampal neurons, OXT early-postnatal treatment has been shown to rescue neurite outgrowth impairment (Reichova et al., 2021) as well as GABA polarity (Bertoni et al., 2021). At the cellular level, examples of OXT effects in neurodevelopmental processes include the regulation of cell fate in neuronal progenitors (Palanisamy et al., 2018), microglia protection against perinatal brain damage (Mairesse et al., 2019), neuron (Ripamonti et al., 2017), and astrocyte differentiation (Alanazi et al., 2020). Autoradiography measures the total level of OXTR in a selected area, but lack the resolution to discriminate binding levels in different cellular populations (i.e., neurons, microglia, astrocytes) and we cannot reveal how the receptor levels varied in these sub-populations in the different areas analyzed. Astrocytes have recently been shown to play a crucial role in mediating positive reinforcement effects of OXT in the central amygdala of mice and rats (Wahis et al., 2021; Baudon et al., 2022) and a reduction in the number of OXTR-expressing astrocytes was found in specific brain regions in another transgenic mouse model of PWS, the Magel2^tm1.1.Stw Mus^ mouse (Althammer et al., 2022). Variations of OXTR levels in astrocytes in early postnatal brain development could crucially contribute to OXT signaling deregulation and life-long effects.

### OXTR levels at P90 in WT and *Magel2*-KO mice

Adult *Magel2*-KO male mice presented a significant up-regulation of OXTR binding levels in the amygdala and hippocampus, indicating an impaired developmental pattern of receptor expression in selected brain regions. Of relevance is our finding that OXT treatment normalized adult OXTR levels in all these regions, consistent with the hypothesis that molecular region-specific effects underlie the behavioral rescue observed in *Magel2* animals treated with OXT at birth (Bertoni et al., 2021). Region-specific OXTR knock-down/rescue experiments will be necessary to show a causal link between OXTR levels in these regions and the expression of specific *Magel2*-KO behavioral phenotypes. Indeed, in mice with a CA2/CA3 region specific OXTR knock-down, the neonatal administration of OXT did not rescue social alterations (Pan et al., 2022) while, in *Dysbindin*-KO mice, a mouse model of schizophrenia with altered OXTR levels in the CeA, the manipulation of OXTR within this region rescued emotional recognition deficits (Ferretti et al., 2019).

An intriguing finding is that in *Magel2*-KO mice, the brain regions responding to postnatal OXT treatment are mainly those displaying dysregulated OXTRs at adulthood. One possible explanation is the existence of a temporal window in which these regions are particularly sensitive to OXT. During the postnatal life, neuronal circuits are undergoing maturation at different times, and under the influence of different external and internal factors. Within these factors, OXT is believed to play a modulatory and integratory role on sensory inputs, allowing to shape brain circuits and connectivity (Grinevich and Stoop, 2018; Muscatelli et al., 2022; Pan et al., 2022). It has been demonstrated that, in mouse pups, sensory experience influences OXT production and that OXT shapes neuronal circuitry by modulating spontaneous and evoked activity (Zheng et al., 2014). In *Magel2-*KO pups, characterized by a deficient production of hypothalamic OXT (Meziane et al., 2015), the neuropeptide may be unable to play its role of mediator of early sensory functions and the postnatal supplementation of OXT may restore this function. Interestingly, in *Magel2-*KO mice, we found a modulation OXT-mediated OXTR in the piriform cortex and amygdala, two structures belonging to a neural circuit that is involved in postnatal learning of the odor-preferences cues crucial to develop social behavior in the early postnatal life (Moriceau and Sullivan, 2005; Oruro et al., 2020).

In the hippocampus we observed a long-lasting effect of OXT treatment, consistent with what we have previously published (Bertoni et al., 2021). We confirm here the OXTR up-regulation in the DG of *Magel2*-KO males and extend the findings of a dysregulation of OXTR also to dCA2/CA3 and vCA2/CA3, indicating a generalized hippocampal involvement. However, while in the DG and dCA2/CA3 we found an up-regulation of OXTR, in the vCA2/CA3 we observed a trend toward receptor down-regulation (statistically significant difference—*p* = 0.029 - in the multiple comparisons in Figure 5A—and close to significance—*p* = 0.081 - in the three-way ANOVA setting in Figure 2). Interestingly, the effect of OXT was selective for the DG and dCA2/CA3, further supporting the idea that the action of OXT is observed only in regions were an up-regulation will manifest in the adult age.

Surprisingly, in *Magel2*-KO mice, we did not find any OXTR dysregulation in a number of regions in which OXTRs are known to play important roles in social and non-social behaviors, such as the LS, MPOA, VMH, and PVT. In particular, in the LS, OXTRs have been shown to be involved in the regulation of different social behaviors including social fear and social preference (Menon et al., 2018); in the MPOA, OXTRs are involved in the modulation of parental (Tsuneoka et al., 2022) and sexual behavior (Menard et al., 2022); OXTRs in the VMH also play an important role in the regulation of sexual behavior (Menard et al., 2022). Lastly, the PVT is a key hub for the control of food seeking and intake and an OXT infusion in this area can attenuate the hypophagia induced by stress and anxiety, with no effects on food intake in normal conditions (Barrett et al., 2021). The lack of an OXTR dysregulation in these regions suggests that they are not the target of OXT in the first postnatal week, when the deficit of OXT production is observed in *Magel2*-KO mice, but will respond to the peptide later on, when the OXT production in the *Magel2-*KO hypothalamus is restored (Meziane et al., 2015). If this is the case, these regions will not suffer from the lack of the peptide in the neonatal period, nor will they be affected from the external OXT administration in the first postnatal week. This is consistent with a delayed modulatory effect of OXT on different brain circuits and functions, and with a lack of OXT neonatal effects on specific circuits/behaviors emerging/consolidating after the first week of life.

In adult *Magel2*-KO females, a completely different trend in OXTR binding levels was found, and an unexpected reduction in OXTR levels was observed in several areas of the brain (Pir, MPOA, LS, MeA, and vCA2/CA3). The functional impact of this receptor down-regulation is at present unclear, given that *Magel2-*KO females did not show impairments in social interactions and learning tests (Meziane et al., 2015). Among these regions, MPOA is an essential component of the neuronal network that regulates parental care and, in rat females, OXT increases sexual receptivity and facilitates maternal behavior (Jurek and Neumann, 2018). Deficits in maternal behavior has been recently reported in *Magel2*-KO females (Bosque Ortiz et al., 2022; Da Prato et al., 2022), however, both these works showed that the delayed maternal pup retrieval was related to reduced USV calls by *Magel2*-deficient pups; assessing studies are needed to evidence deficits in sexual/parental behavior that may be correlated to the MPOA OXTR alterations observed in *Magel2-KO* females.

Even more surprisingly, we found a further down-regulation of OXTR binding sites in OXT-treated adult females in the VMH, PVT, and dCA2/CA3. This may represent a compensatory mechanism to protect these regions from an overload of OXT in the first postnatal week, to which only specific females’ regions would be sensitive. It will be interesting to assess if this compensatory OXTR down-regulation protects the female brain from behavioral alterations by pharmacological/genetic manipulation of OXTR in these areas of Magel2*-KO* females.

### Sexual dimorphism in WT and *Magel2*-KO mice

The OXT/OXTR system, together with the highly related vasopressin system, have been long known to be sexually dimorphic (de Vries, 2008; Carter et al., 2020). For example, in humans, following a social stress test, a single dose of intranasal OXT increases distress and anger in women, but reduces distress in men (Kubzansky et al., 2012). In mice, activation of OXT interneurons in the medial prefrontal cortex (mPFC) is anxiolytic in males and prosocial in females (Nakajima et al., 2014; Li et al., 2016), while in California mice (*Peromyscus californicus*), a rodent species in which females regularly engage in territorial aggression similarly to males, activation of OXT neurons in the bed nucleus of the stria terminalis (BNST) has sex-specific effects on social approach, social avoidance and social vigilance (Duque-Wilckens et al., 2018; Williams et al., 2020). In the rat, sex-specific differences in OXTR distribution have been extensively investigated (Smith et al., 2017; Bredewold and Veenema, 2018), but only few studies are present in mice.

At P8, we did not observe sex-dependent differences in binding levels of OXTR in WT or *Magel2*-KO animals, suggesting that male/female differences in OXTR brain levels arise later in the postnatal life, possibly as a consequence of sexual hormonal influences (Ivell and Walther, 1999; Champagne et al., 2001). At this early postnatal developmental stage, OXT seems to exert general and crucial effects, with an impact on survival, such as those on suckling activity, which is impaired in both male and female *Magel2*-KO pups (Schaller et al., 2010; Meziane et al., 2015).

Adult WT mice presented sex-specific differences in OXTR expression in almost all regions analyzed, with OXTR levels always higher in females, in comparison to another study in which multiple brain regions of male and female mice were analyzed and a significant sexual dimorphism was found only in two hypothalamic regions, the ventral premammillary nucleus and the anteroventral periventricular nucleus (Newmaster et al., 2020), however, in the latter study the OXTR gene expression was determined in a transgenic *knock in* line by measuring a cytoplasmic Venus-GFP reporter that may not exactly match the level of membrane OXTR binding sites. A study in which OXTRs were evaluated with an anti-OXTR antibody, reported increased levels of OXTR in CeA, CA2, LS, Pir, and VMH of virgin females as compared to males (Mitre et al., 2016), in agreement with our current findings.

Most importantly, the sexual dimorphism was greatly impaired in adult *Magel2*-KO mice which only presented a sex-dependent difference in OXTR levels in the VMH and in the Pir, the latter region presenting a higher expression of OXTR levels in males. Further studies are needed to assess the role played by OXTR in regulating *Magel2* specific behaviors in these regions.

Our present findings confirm sex-based differences in OXTR levels, highlighting the need for an extensive investigation of the specific role of OXT and OXTR in the female mouse brain, in particular in preclinical models of neurodevelopmental disease characterized by a well-recognized sex-bias.

## Conclusion

The present study underscores the relevance of long-lasting sex and region-specific effects of postnatal OXT treatment on OXTR levels in the *Magel2*-KO mouse model of the PWS/SYS. Understanding the cellular and molecular mechanisms of this specific OXTR regulation represents a crucial step toward developing effective OXT-based therapeutic strategies.

## Limitations of this study

A limitation of the present study is the absence of WT-OXT treated groups in our experimental design, as investigating the role of OXT treatment in WT animals was outside the main scope of this work. This study could also have been extended to include control groups of “handled only” mice (i.e., manipulated pups not receiving any injection) as it is well established that pup handling can influence OXTR levels in the brain (Bales and Perkeybile, 2012). In our experimental conditions, vehicle and OXT-treated pups were similarly handled, most likely leveling handling contribution equally across the groups. Particular attention was placed on WT mice used as controls, which all received postnatal vehicle injections, representing, in our opinion, the most appropriate controls to selectively highlight postnatal OXT-induced long-lasting effects on OXTR. The “non-handled” extra control groups would have contributed to build a wider and deeper knowledge of the physiological developmental changes in OXTR expression and distribution in the brain. However, the main aim of this study was to investigate the effect of early postnatal OXT treatment in male and female *Magel2*-KO mice in order to advance the translational research on OXT treatment for Schaaf-Yang Syndrome and Prader-Willi patients. Pilot clinical studies of OXT treatment in PWS and SYS infants have provided encouraging positive results (Tauber et al., 2017) and strong preclinical evidence on the effects of OXT treatment in *Magel2*-deficient animal models on biochemical and molecular parameters, including OXTR levels in the brain, is urgently needed.

## Data availability statement

The original contributions presented in this study are included in the article/Supplementary material, further inquiries can be directed to the corresponding authors.

## Ethics statement

The animal study was reviewed and approved by French Ministry of Agriculture with the accreditation no. B13-055-19.

## Author contributions

FSa and AB performed the experiments and analyzed the data. VG analyzed the data, prepared the figures, and contributed to the preparation of the manuscript. MB analyzed the data, prepared the figures, and contributed to the writing of the manuscript. CP analyzed the data. FSc generated the mice and collected brains. FM contributed to the conception of the project. FSc and AB supervised the project. BC contributed to the conception of the project, supervised the planning and execution of the experiments, collected and analyzed the data, and prepared the manuscript. All authors supervised the manuscript and approved the submitted version of the manuscript.

## Footnotes

1
http://www.gpower.hhu.de
