# Supplementary figures and images for: Oxytocin receptors in the Magel2 mouse model of autism: Specific region, age, sex and oxytocin treatment effects
Source: Front Neurosci. 2023 Mar 14;17:1026939. doi: 10.3389/fnins.2023.1026939 (PMC10043208; doi:10.3389/fnins.2023.1026939)

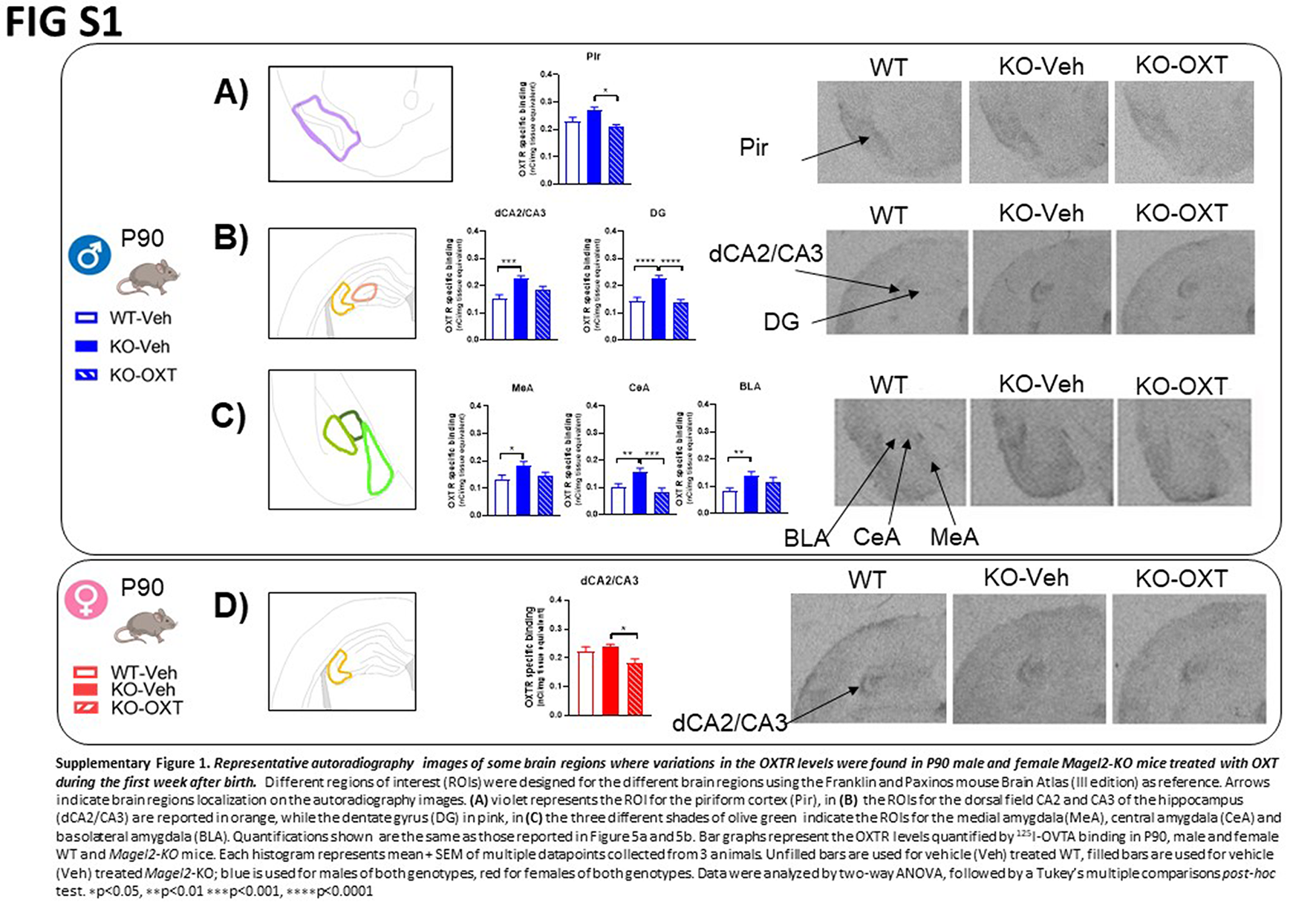

Supplement: Supplementary file 1 [file Image_1.tif]
